# Laminin-1-induced migration of multiple myeloma cells involves the high-affinity 67 kD laminin receptor

**DOI:** 10.1054/bjoc.2001.2078

**Published:** 2001-09-01

**Authors:** I Vande Broek, K Vanderkerken, C De Greef, K Asosingh, N Straetmans, B Van Camp, I Van Riet

**Affiliations:** Department of Hematology and Immunology, Free University Brussels (VUB), Laarbeeklaan 101, Brussels, B-1090, Belgium; Department of Internal Medicine and Hematology, Saint-Luc Hospital, Brussels, Belgium

**Keywords:** multiple myeloma, homing, laminin-1, 67LR, migration, extravasation

## Abstract

The 67 kD laminin receptor (67LR) binds laminin-1 (LN), major component of the basement membrane, with high affinity. In this study, we demonstrated that human multiple myeloma cell lines (HMCL) and murine 5T2MM cells express 67LR. CD38^bright+^ plasma cells in fresh multiple myeloma (MM) bone marrow (BM) samples showed weaker 67LR expression, but expression increased after direct exposure to a BM endothelial cell line (4LHBMEC). LN stimulated the in vitro migration of 3 HMCL (MM5.1, U266 and MMS.1), primary MM cells and the murine 5T2MM cells. 67LR has been shown to mediate the actions of LN through binding to CDPGYIGSR, a 9 amino acid sequence from the B1 chain of LN. MM cell migration was partially blocked by peptide 11, a synthetic nonapeptide derived from this amino sequence and also by a blocking antiserum against 67LR. Co-injection of peptide 11 with 5T2MM cells in a murine in vivo model of MM resulted in a decreased homing of 5T2MM cells to the BM compartment. In conclusion, LN acts as a chemoattractant for MM cells by interaction with 67LR. This interaction might be important during extravasation of circulating MM cells. © 2001 Cancer Research Campaign


*British Journal of Cancer* (2001) **85**(9), 1387–1395
© 2001 Cancer Research Campaign
doi: 10.1054/ bjoc.2001.2078, available online at http://www.idealibrary.com on IDEAL

http://www.bjcancer.com

### Laminin-1-induced migration of multiple myeloma cells involves the high-affinity 67 kD laminin receptor

**I Vande Broek[1], K Vanderkerken[1], C De Greef[1], K Asosingh[1], N Straetmans[2], B Van Camp[1] and I Van Riet[1]**

[1]Department of Hematology and Immunology, Free University Brussels (VUB), Laarbeeklaan 101, B-1090, Brussels, Belgium
[2]Department of Internal Medicine and Hematology, Saint-Luc Hospital, Brussels, Belgium

**Summary** The 67 kD laminin receptor (67LR) binds laminin-1 (LN), major component of the basement membrane, with high affinity. In this study, we demonstrated that human multiple myeloma cell lines (HMCL) and murine 5T2MM cells express 67LR. CD38$^{bright+}$ plasma cells in fresh multiple myeloma (MM) bone marrow (BM) samples showed weaker 67LR expression, but expression increased after direct exposure to a BM endothelial cell line (4LHBMEC). LN stimulated the in vitro migration of 3 HMCL (MM5.1, U266 and MMS.1), primary MM cells and the murine 5T2MM cells. 67LR has been shown to mediate the actions of LN through binding to CDPGYIGSR, a 9 amino acid sequence from the B1 chain of LN. MM cell migration was partially blocked by peptide 11, a synthetic nonapeptide derived from this amino sequence and also by a blocking antiserum against 67LR. Co-injection of peptide 11 with 5T2MM cells in a murine in vivo model of MM resulted in a decreased homing of 5T2MM cells to the BM compartment. In conclusion, LN acts as a chemoattractant for MM cells by interaction with 67LR. This interaction might be important during extravasation of circulating MM cells. © 2001 Cancer Research Campaign

**Keywords**: multiple myeloma; homing; laminin-1; 67LR; migration; extravasation

Multiple myeloma (MM) represents a B cell malignancy, characterised by a monoclonal proliferation of malignant plasma cells (Ludwig et al, 1999). During the main course of disease evolution, terminally differentiated B cells preferentially accumulate in the bone marrow (BM) and represent the main cell type. However, several studies indicated that the tumour clone also includes circulating B cells that might represent the 'myeloma stem cell'. Clear evidence supporting this statement was provided by molecular techniques showing that MM patients have B cells in circulation harbouring the same immunoglobulin (Ig) gene rearrangements as the malignant BM plasma cells (Van Riet et al, 1989). Moreover it was demonstrated that these circulating MM cells show in their Ig genes the presence of somatic mutations without intraclonal variation (Bakkus et al, 1992). This implicates that MM originates from a B cell that has been antigen-selected in the germinal centre of the lymph node. The restricted localisation of MM cells in the BM during the initial stage of disease could be explained by a selective initial homing of circulating MM cells to the BM microenvironment and/or by the presence of a unique combination of survival and growth factors provided by the BM microenvironment not present in other tissues (Van Riet et al, 1998; Vanderkerken et al, 2000).

In analogy to the migration mechanisms used by normal leukocytes and metastatic carcinoma cells (Liotta, 1986; Butcher and Picker, 1996), the migration of MM cells to the BM is likely to be mediated by similar mechanisms. During lymphocyte homing and tumour cell invasion, cells attach to and degrade components of the extracellular matrix (ECM) before passing through. Previous studies already revealed the importance of interactions of malignant cells with the basement membrane during invasion. The basement membrane represents a specialised form of ECM, which separates the endothelium from the underlying interstitial stroma. It is composed of several components including collagen IV, glycoproteins such as laminin, fibronectin and proteoglycans (Vracko, 1974). Components of the ECM, including laminin, fibronectin and type IV collagen, as well as their proteolytic digestion products have been shown to stimulate the in vitro migration of a variety of normal and malignant cell types (Klominek et al, 1993). Laminin-1 (LN), a well characterised cell adhesion protein, represents the major non-collagenous glycoprotein of the basement membrane, involved in multiple important biological activities such as cell adhesion, migration, spreading, proliferation, growth and differentiation (Malinda and Kleinman, 1996). Very recently, Spessotto et al (2001) have demonstrated that different LN isoforms may evoke diverse cellular responses in different neoplastic lymphocytes. Invasion of the subendothelial basement membrane is mediated in part by interactions of tumour cells with laminin via specific cell surface receptors (Ramos et al, 1991). The 67 kD laminin-binding protein (67LR) represents a non-integrin-binding protein which binds LN with high affinity (Malinoff and Wicha, 1983). Expression of 67LR has been shown to be increased in neoplastic cells as compared to their normal counterparts and directly correlates with an enhanced invasive and metastatic potential (D'Errico et al, 1991; Kim et al, 1998). Additionally, suppression of the expression of the precursor of 67LR (37LBP) with antisense 37LBP/p40 RNA using a retroviral vector reduces the capability of lung cancer cell proliferation in vitro and tumour formation in vivo (Satoh et al, 1999), clearly indicating the role of 67LR in tumorigenicity.

In the present study, we demonstrated that MM cells express the 67 kD high-affinity LN receptor. In addition, we found that LN acts as a chemoattractant for MM cells by binding to 67LR.

*Received 21 February 2001*
*Revised 17 July 2001*
*Accepted 23 July 2001*

*Correspondence to:* I Van Riet

### MATERIALS AND METHODS

### Human MM cell lines (HMCL)

Three well characterised HMCL (MM5.1, MMS.1 and U266) were selected for our experiments. They were cultured as described (Nilson, 1971; Okuno et al, 1991; Van Riet et al, 1997).

### Patient samples

BM samples from 5 MM patients (pts) (age 52–94, mean 66.5; M/F, 3/5) were collected during standard diagnostic procedures. The study was approved by the local ethical committee. According to Durie and Salmon's clinical staging system (1975), patients were distributed as follows: stage I, 1 pt; stage II, 2 pts; stage III, 2 pts. BM aspirates were collected in a heparinised syringe. Mononuclear cells were separated by density gradient centrifugation over Ficoll-Hypaque (Life Technologies).

### MACS separation of primary MM cells

Primary MM cells expressing the CD138 antigen were immunomagnetically separated using the Magnetic Cell Sorting System (MACS) (Miltenyi Biotech). Cells were incubated for 15 minutes at 4˚C with MACS microbeads conjugated to a monoclonal mouse CD138 (syndecan-1) antibody (clone B-B4, isotype mouse IgG1) (40 µl $10^7$ cells). Cells were washed once in PBS supplemented with human albumin (4%), resuspended and separated on a column placed in the magnetic field of the MACS separator. CD138+ cells were retained in the column and eluted as positively selected cell fraction after removal of the column from the magnetic field. Cells were counted and viability was assessed with trypan blue. MACS purification produced a ≥ 97% pure preparation.

### 5T2 murine MM model

Male C57B1/KaLwRijHsd mice, 6–10 weeks old were purchased from Harlan CPB. Mice were housed under conventional conditions and fed ad libitum (license number LA 1230281). 5T2MM cells originated spontaneously in aging mice (Radl et al, 1979). The 5T2MM model displays characteristics analogous to human MM (Asosingh et al, 2000). Cells were prepared as described previously (Vanderkerken et al, 1997). Briefly, cells were obtained by flushing the BM of hind legs. Cells were purified by gradient centrifugation on lymphocyte M (Cedarlane), washed and purified by Percoll (Pharmacia AB) gradient centrifugation. Cell purity (> 90%) was verified by FACS staining with 5T2MM anti-idiotype specific antibodies (Vanderkerken et al, 1997). 5T2MM cells were used for in vitro and in vivo experimental assays.

### Peptides

LN, extracted and purified by immunoaffinity from the Engelbreth-Holm-Swarm (EHS) tumour was purchased from Sigma-Aldrich and peptide 11, a synthetic nonapeptide derived from the B1 chain of LN was purchased from Eurobiochem. A control scrambled nonapeptide (Ala-Asp-Cys-Gly-Gly-Ile-Pro-Ser-Tyr) was purchased from Genosys.

### RNA extraction and Northern blotting

Northern blot hybridisation has been used to assess the expression of mRNA for the 37 kD precursor of 67LR from total RNA extracts from cultured HMCL. The plasmid DNA which carries the cDNA for the 37 kD precursor of 67LR (a kind gift from Dr Vincent Castronovo, Mestastasis Research Laboratory, University of Liege, Belgium) was digested with XbaI. The released fragment was purified using the Wizard DNA clean up purification kit (Promega). Total RNA was isolated from cultured HMCL with the RNeasy Mini Kit (Qiagen). For Northern blot analysis, equal amounts of total RNA (10 µg) were denatured with 37% formaldehyde and deionised formamide, subsequently separated through a 1% agarose-formaldehyde gel and blotted onto Biobond nylon membranes (Sigma-Aldrich). cDNA probes encoding for the 37 kD precursor of 67LR were radioactively labelled with $^{32}$P-dCTP. Prehybridisation was performed in 50% formamide, 5 × Denhardt's solution, 2 × SSC, 1% SDS and 100 mg $ml^{-1}$ denatured salmon sperm DNA for 3 hours at 42˚C. Blots were subsequently hybridised overnight at 42˚C. After hybridisation, membranes were stringently washed by vigorous rotation at 42˚C for respectively 5, and 2 × 30 minutes in each of the following solutions: 2 × SSC/0.1% SDS and 0.2 × SSC/0.1% SDS. After washing, membranes were exposed to film (Kodak Biomax MR) overnight at –80˚C. As a control for the quality and quantity of the RNA samples analysed, the same blots were hybridised with a glyceraldehyde-3-phosphate dehydrogenase (GAPDH) cDNA probe. The blotting experiment was repeated twice with different RNA preparations.

### Flow cytometric analyses

Fluorescence-activated cell sorting (FACS) analyses were performed to characterise the expression of 67LR on HMCL. After washing, cells were incubated with a monoclonal antibody (MLuC5) (IgM) recognising 67LR (kindly provided by Dr V Castronovo, Metastasis Research Laboratory, University of Liege, Belgium) and a polyclonal rabbit antiserum against 67LR (provided by Dr HK Kleinman, Institute of Dental and Craniofacial Research, National Institute of Health, Bethesda, USA). The last mentioned antiserum was also used to demonstrate 67LR expression on the murine 5T2MM cell line. As negative control, cells were incubated with isotype-matched irrelevant mouse Ig or rabbit Ig antibodies. Cells were subsequently washed in PBS/BSA and incubated with rabbit anti-mouse Ig antiserum or swine anti-rabbit Ig antiserum, both conjugated with fluorescein isothiocyanate (FITC) (Dako). Next, cells were washed, resuspended in PBS/BSA and finally analysed by flow cytometry (EPICS XL, Coulter Electronics). 67LR expression on MM plasma cells from patient samples was evaluated by a double staining procedure. Mononuclear cells isolated by Ficoll centrifugation were incubated with the MLuC5 monoclonal antibody as indicated above. After washing, cells were simultaneously incubated with a PE conjugated goat anti-mouse IgM antiserum (Southern Biotechnology) and a Cy-5-conjugated CD38 specific antibody (HIT2) (BD Pharmingen). Plasma cell identification was controlled by co-expression of CD38 and intracytoplasmic κ/λ immunofluorescence using three-colour staining (rabbit anti-human Igκ-FITC and Igλ-PE $F(ab)_2$ fragments (Dako)).

 *© 2001 Cancer Research Campaign*

### Co-culture of MM plasma cells with an endothelial cell line

In order to evaluate whether the expression of 67LR on MM plasma cells could be modulated by exposure to endothelial cells, mononuclear cells isolated from MM BM samples were co-cultured for 24 hours with the BM endothelial cell line 4LHBMEC (kindly provided by Dr A Dräger, Department of Hematology, Free University Amsterdam, The Netherlands). This cell line was cultured in 10% FCS medium 199 with 10 ng ml$^{-1}$ ECGF (Roche) until a confluent adherent mono cell layer was obtained. Mononuclear cells were incubated at $10^6$ cells ml$^{-1}$ in 5% FCS-RPMI with or without pre-established confluent monolayers of 4LHBMEC. After 24 hours non-adherent cells were removed by gentle pipeting and FACS analysis was performed.

### In vitro cell migration assays

We performed in vitro migration assays with murine and human MM cells using an in vitro Transwell® cell migration system (Costar Corning) with 5 and 8 µm pore size membrane filters respectively. MM cells ($1 \times 10^5$) were added to the upper well in 100 µl medium. LN was diluted in 300 µl RPMI 1640 medium in varying concentrations and added to lower wells. Cells were tested for their spontaneous motility in response to control medium (RPMI 1640). The migration responses to LN were tested with controls in the same assay. After an incubation of 3 hours at 37˚C in a humidified 5% $CO_2$ atmosphere, the number of cells that migrated through the filter into lower wells was quantified with a non-radioactive, colorimetric assay system using XTT (Roche) (Roehm et al, 1991). XTT with phenazine methosulfate (PMS) was added in each well after removal of inserts and incubated for 5 hours at 37˚C. The absorbance of converted stain was measured spectrophotometrically with a 96-well microplate reader (Ceres 900 Bio-Tek International Inc) at a wavelength of 450 nm with a reference wavelength of 650 nm. Migration responses were determined as the percentage increase in cell migration compared to spontaneous migration. All experiments were performed in triplicate. Zigmund–Hirsh checkerboard analysis (Zigmund and Hirsch, 1973) was performed to distinguish chemokinesis from chemotaxis. For migration-inhibition experiments, MM cells were incubated with peptide 11 or with a blocking polyclonal antiserum directed against 67LR (Clement et al, 1990). Scrambled peptide 11 and a polyclonal rabbit IgG (Chemicon International) served as controls. LN was used at 50 µg ml$^{-1}$. Migration responses were compared with control migration to LN and determined as described above.

For transendothelial migration, $2 \times 10^5$ endothelial cells (4LHBMEC) were seeded on Transwell$^R$ filters. Confluent monolayers were formed after two days. Freshly isolated primary MM cells ($1 \times 10^5$) were added to the upper compartment of the Transwell$^R$ system, with or without peptide 11 (50 µg ml$^{-1}$), and allowed to migrate overnight. LN (50 µg ml$^{-1}$) was added as chemoattractant to the lower compartment. Transmigrated MM cells were recovered and counted by flow cytometry (FACSort, Becton Dickinson). Migration responses were determined as described.

### In vivo experimental assays

For in vivo experiments, 5T2MM cells were labelled with 500 µCi $^{51}$Cr-chromate (Amersham). After washing, cell viability was assessed by trypan blue exclusion and cells were resuspended in RPMI 1640 at $2.5 \times 10^6$ ml$^{-1}$. Peptide 11 or scrambled peptide 11 was solubilised at 2 mg ml$^{-1}$ in PBS and mixed with 5T2MM cells suspensions ($5 \times 10^5$) in a final volume of 200 µl. After 5 min incubation, 5T2MM cells were co-injected with peptide 11, scrambled peptide 11 or PBS intravenously into the tail vein of syngeneic mice. After 18 hours, mice were killed, legs, ribs and vertebrae were removed, subsequently rinsed in PBS and the radioactivity was determined by gamma-counting (MR48OC/TP ITM, Van Hopplynus) to estimate the level of tumour infiltration in the BM (Vanderkerken et al, 2000). Control and treatment groups each contained 9 mice.

### Statistical analysis

Statistical analyses were performed using the 2-sample *t*-test. A *P* value of less than 0.05 was considered significant. Values were expressed as the mean ± SD.

### RESULTS

### LN receptor expression by HMCL, murine 5T2MM cells and fresh MM plasma cells

To characterise the HMCL with respect to their expression of 67LR, we performed Northern blotting and flow cytometric analysis using a specific monoclonal antibody to 67LR. Northern blot analysis revealed a homogeneous expression of mRNA for the 37 kD precursor of 67LR in all 3 HMCL tested (Figure 1A). Flow cytometry demonstrated the surface expression of 67LR on HMCL and the murine 5T2MM cells (Figure 1B). 67LR expression on CD38$^{bright+}$ plasma cells from 5 MM BM samples was more weak (Figure 1B). However, an increased expression of this receptor could be observed after direct exposure of tumour cells to a BM endothelial cell line (4LHBMEC) as illustrated in 3 patients (Figure 2).

### Effect of LN on the in vitro migration of HMCL and murine 5T2MM cells

The migration of 3 HMCL (MM5.1, U266 and MMS.1) and the 5T2MM cells was evaluated in the presence of LN. As shown in Figure 3, increasing concentrations of LN (10 to 100 µg ml$^{-1}$) resulted in an increased number of cells migrating through the microporous membrane, with a mean increased migration response up to 90%. Checkerboard analyses revealed that MM cell migration to LN was chemotactic with a chemokinetic component (Table 1).

### Inhibitory effect of peptide 11 on MM cell migration to LN

Peptide 11 has been shown to possess the ability to inhibit the biological activity of LN by competing for binding with 67LR. We investigated whether peptide 11 could modulate the interactions between MM cells and LN and we tested its effect on the migration of MM5.1 and 5T2MM cells in response to LN. In vitro cell migration assays were carried out with LN (50 µg ml$^{-1}$) added to the lower well of the migration system. MM cells were placed in upper wells, together with peptide 11 (10, 50 or 100 µg ml$^{-1}$) or scrambled peptide 11 (100 µg ml$^{-1}$). As shown in Figure 4, peptide 11 inhibited MM cell migration to LN.

© 2001 Cancer Research Campaign 

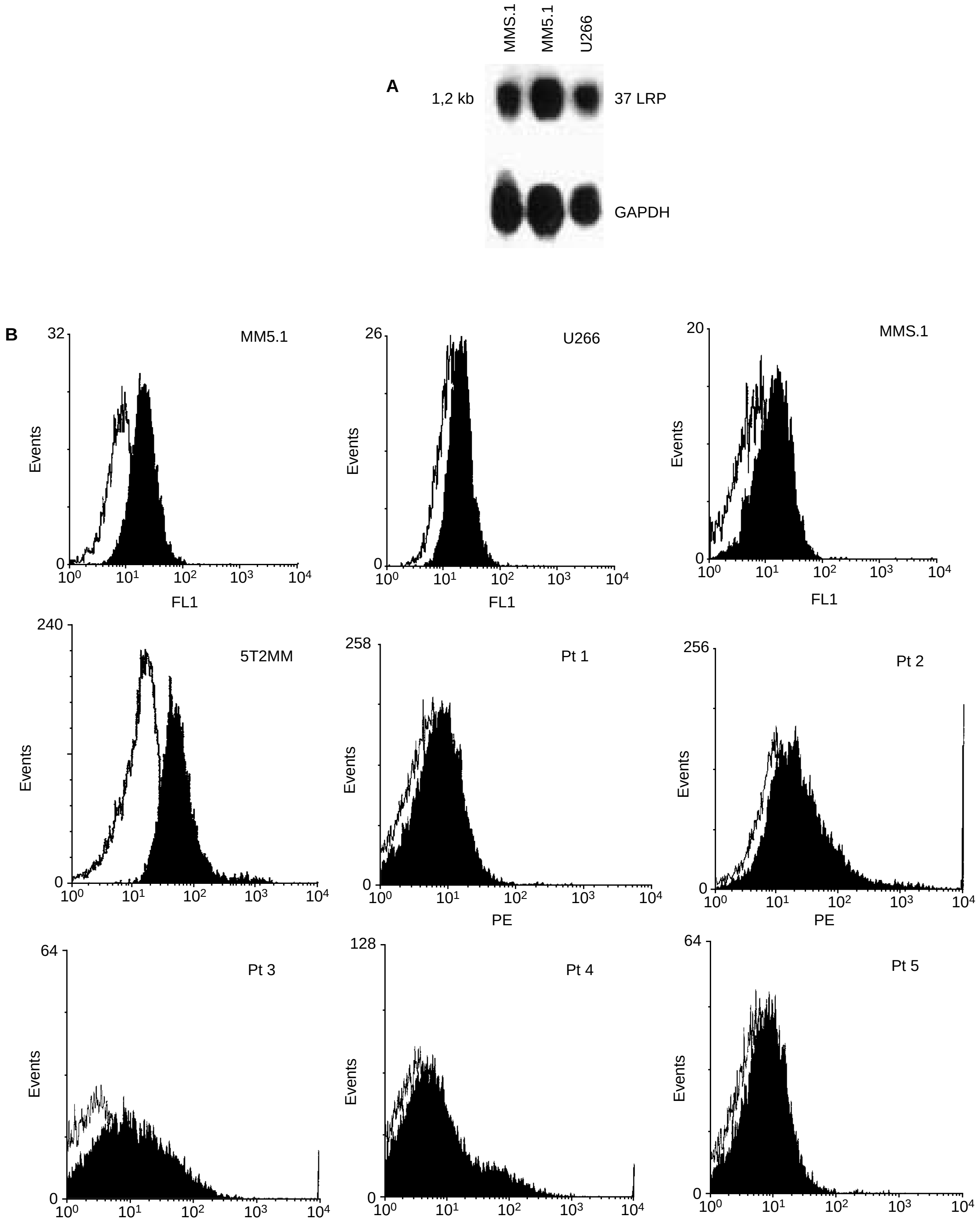

**Figure 1** Expression of 67LR on HMCL, murine 5T2MM cells and MM cells from BM samples. (**A**) Representative Northern blot signals to transcripts from three HMCL show a homogeneous expression of the 37 kD precursor of 67LR in all tested HMCL. (**B**) Representative results of flow cytometric analysis are shown and expressed as log fluorescence versus number of cells. Background fluorescence is represented by white histograms, 67LR expression by black histograms. 67LR expression on malignant plasma cells from five bone marrow samples of MM patients was analysed by two-colour immunofluorescence using CD38-Cy5

However, with increasing concentrations of peptide 11, inhibition of cell migration was gradually reduced. This last effect most likely relates to the chemokinetic effect of peptide 11 (Graf et al, 1987a). Scrambled peptide 11 did not affect in vitro MM cell migration to LN.

### Effect of a blocking antiserum against 67LR on MM cell migration to LN

In order to evaluate the importance of 67LR in the interaction of MM cells with LN, we tested the effect of a blocking polyclonal

 *© 2001 Cancer Research Campaign*

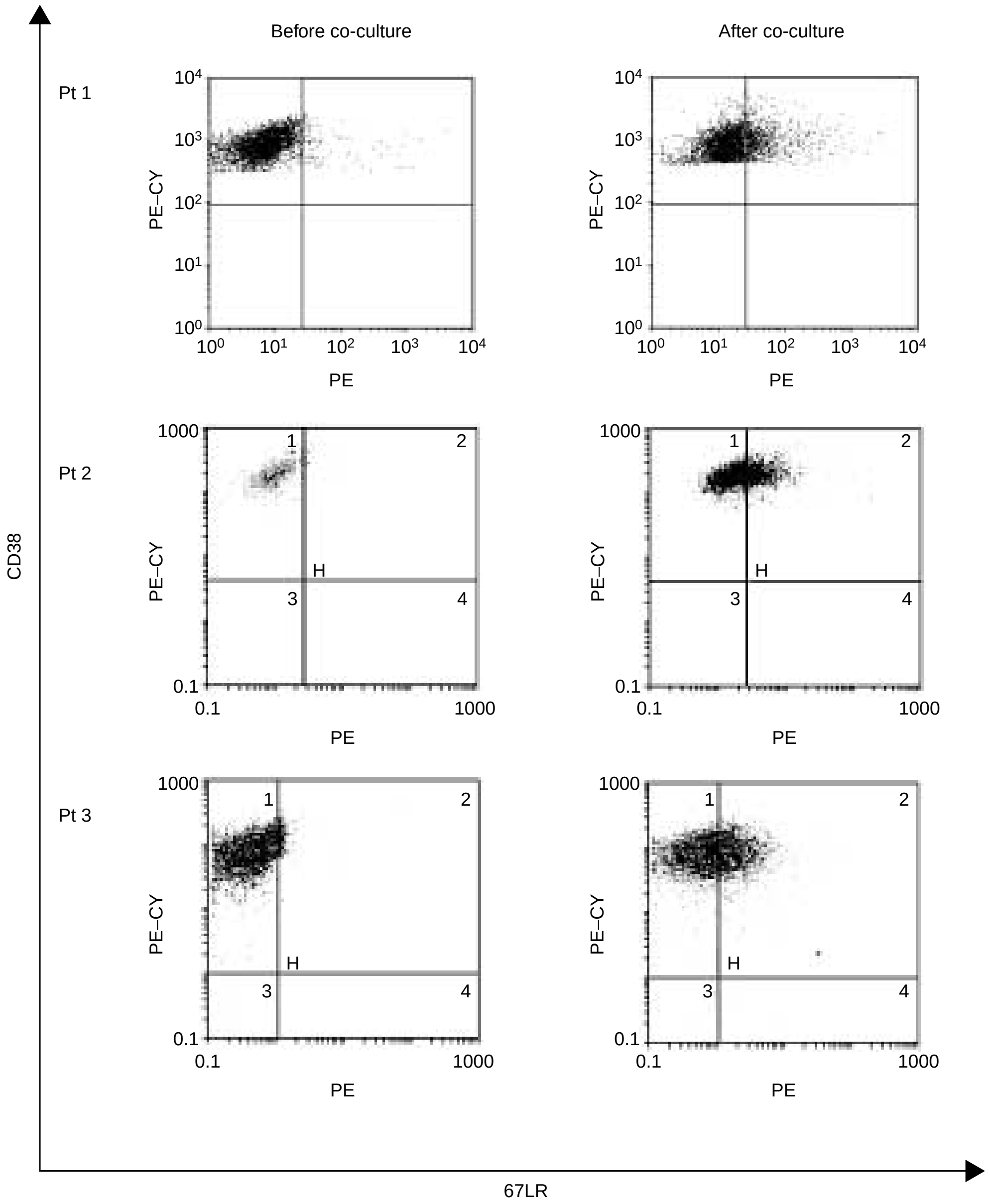

**Figure 2** Modulation of 67LR expression on MM plasma cells after exposure to the BM endothelial cell line 4LHBMEC. 67LR expression on malignant plasma cells was analysed before and after co-culturing mononuclear cells from three MM bone marrow samples with the 4LHBMEC cell line. Cells were analysed by two-colour immunofluorescence using CD38-Cy5

antiserum against 67LR on MM cell migration to LN. As shown in Figure 5, antiserum against 67LR partially inhibited the migration response induced by LN in a dose-dependent manner.

### Effect of LN and peptide 11 on the in vitro migration of primary MM cells

Previously, in vitro cell migration experiments to laminin were performed using primary MM cells freshly isolated from the bone marrow of patients with myeloma. Only minor migration responses were observed when LN was added to the lower compartment (data not shown). Considering the weak basal expression of 67LR on primary MM cells and its up-regulation when co-cultured with bone marrow endothelium, we carried out in vitro migration assays after coating the Transwell$^R$ filters with BM endothelial cells. In this condition, a significant migration response was observed to LN, indicating that 67LR was up-regulated by contact with endothelium, as found also in the co-culture experiments. LN-induced migration of primary MM cells could also be inhibited by peptide 11, again suggesting a role of 67LR in the observed migration responses (Fig. 6).

### Effect of peptide 11 on the in vivo migration of murine MM cells

The 5T2MM murine model was also used to evaluate whether treatment with peptide 11 affects the in vivo migration of tumour cells to the BM. Therefore, we injected $^{51}$Cr-labelled 5T2MM cells

*© 2001 Cancer Research Campaign* 

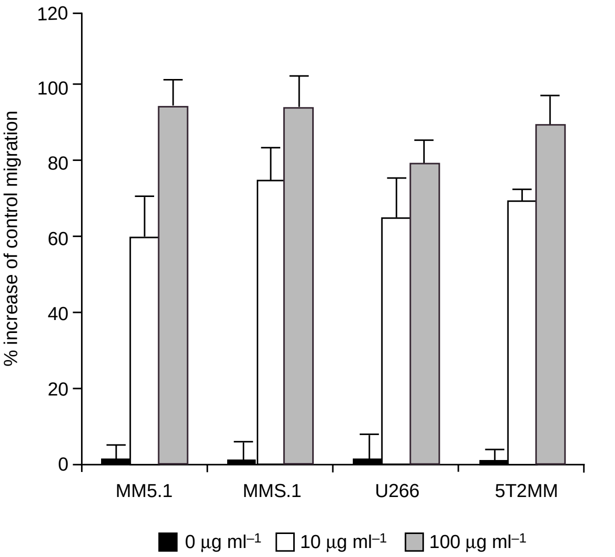

**Figure 3** Migration of HMCL and murine 5T2MM cells in the presence of LN. LN was tested for its ability to promote the migration of HMCL and 5T2MM cells. Increasing concentrations of LN were added to the lower wells of the Transwell® migration system as indicated. As negative control, dilution medium (RPMI) was added to lower wells. Columns represent the percentage increase of control migration in the presence of various amounts of peptide as indicated. Mean increase of control migration ± SD of 3 independent experiments is shown

**Table 1** Checkerboard analysis of MM5.1 cell migration to laminin

| **% increase of control migration** | **0 µg ml$^{-1}$** | **10 µg ml$^{-1}$** | **50 µg ml$^{-1}$** | **100 µg ml$^{-1}$** |
|---|---|---|---|---|
| 0 µg ml$^{-1}$ | 1 | 1 | 1 | 1 |
| 10 µg ml$^{-1}$ | 5 | 16 | 53 | 53 |
| 50 µg ml$^{-1}$ | 32 | 36 | 63 | 73 |
| 100 µg ml$^{-1}$ | 68 | 73 | 68 | 52 |

MM5.1 cells were analysed for their motility response to different concentrations of laminin (0–100 µg ml$^{-1}$) added to upper and lower wells of the Transwell migration system. Each square of the 'checkerboard' represents a condition with a unique gradient. The number in each square represents the cell migration in response to the given concentration of attractant in upper and lower wells (chemotaxis). The diagonal shows the number of cells which migrated in the absence of gradient (chemokinesis). Migration responses are presented as the percentage increase of control migration to RPMI. Representative results of three independent assays.

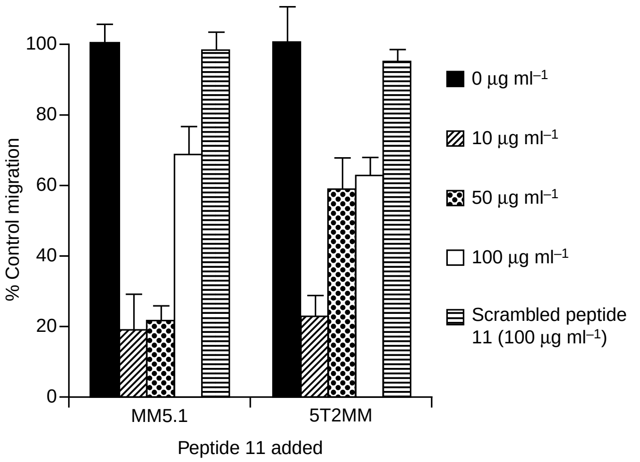

**Figure 4** Effect of peptide 11 in inhibiting MM cell migration to LN. In vitro cell migration assays were performed with LN added into lower wells at 50 µg ml–$^{1}$. MM5.1 and murine 5T2MM cells were placed into upper wells, together with various concentrations of peptide 11 or scrambled peptide 11 as indicated. Inhibition is represented as the mean percentage of cell migration (± SD) compared to control MM cell migration (100%) towards LN. Experiments were repeated twice

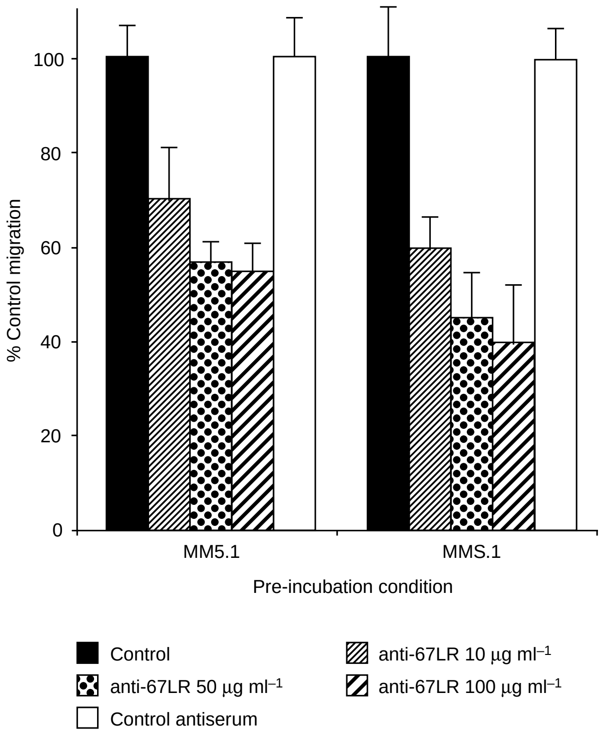

**Figure 5** Effect of a polyclonal blocking antiserum against 67LR on MM cell migration to LN. Cell migration of 2 HMCL is shown (MM5.1 and MMS.1). LN was used at 50 µg ml$^{-1}$. MM cells were incubated with various concentrations of a polyclonal blocking antiserum against 67LR and a control rabbit IgG antiserum as indicated. Controls were incubated without antisera. Inhibition is represented as the mean percentage of cell migration (± SD) compared to control MM cell migration (100%) towards LN. Experiments were repeated twice together with peptide 11 or scrambled peptide 11 intravenously into the tail vein of syngeneic mice. Tumour cell infiltration in the BM was determined by counting the radioactivity in legs, ribs and vertebrae after 18 hours. Results were represented as the % of recovered cpm in control and treated animals. As shown in Figure 7, co-injection of 5T2MM cells with peptide 11 significantly reduced (25%, $P < 0.05$) MM cell migration to the BM compartment. Scrambled peptide 11 did not affect the in vivo MM cell migration.

### DISCUSSION

The homing of circulating MM cells to the BM implicates that these cells must be equipped with appropriate cell surface receptors, which allow binding to and traversing through the endothelium. Very little information is available about the molecular mechanisms used by MM cells during extravasation. Several studies indicated that the interaction of tumour cells with components of the ECM represents a key step in the biology of cancer cell migration and invasion. In the present study, we focused on the specific role of LN in MM cell migration. 3 well-characterised

 © 2001 Cancer Research Campaign

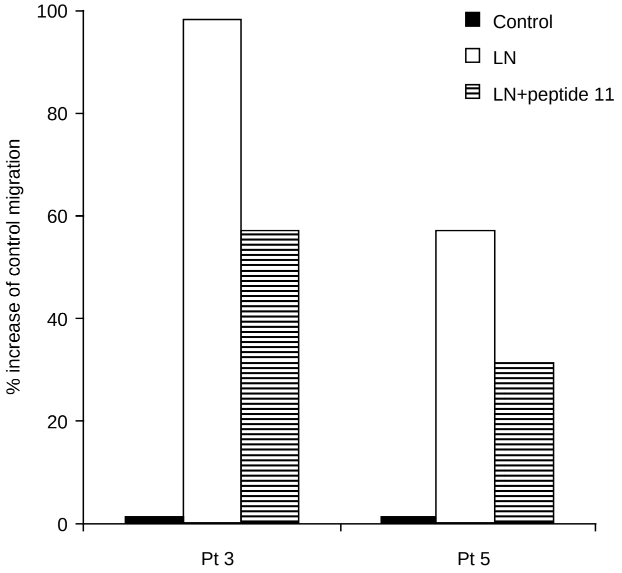

**Figure 6** Effect of LN on the in vitro transendothelial migration of primary MM cells. For transendothelial cell migration, Transwell[R] filters were coated with bone marrow endothelial cells as described in Materials and Methods. LN was added into lower wells at 50 μg ml$^{-1}$. As negative control, dilution medium (RPMI) was added to lower wells. Freshly isolated myeloma plasma cells from 2 patients (pt 3 and pt 5) were placed into upper wells, with or without peptide 11 (50 μg ml$^{-1}$). Columns represent the percentage increase of control migration

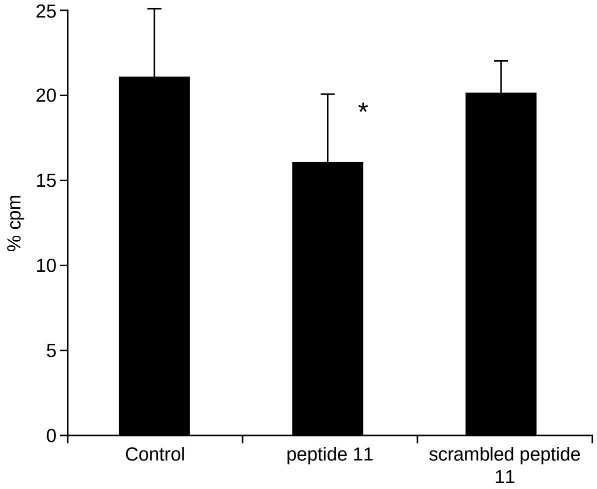

**Figure 7** Effect of peptide 11 on the in vivo migration of 5T2 MM cells. 5T2MM cells were labeled with $^{51}$Cr, mixed with peptide 11 (2 mg ml$^{-1}$) or scrambled peptide 11 and injected intravenously into the tail vein of syngeneic mice. After 18 hours, mice were killed; legs, ribs and vertebrae were removed, subsequently rinsed in PBS and radioactivity was determined by gamma counting. Control and treatment groups contained 8 mice. Each column represents the mean percentage ± SD of total recovered radioactivity in the bone marrow. The *P* value was calculated from the 2- sample *t*-test (*: statistically significant)

HMCL, as well as the murine 5T2MM cell line were selected for our experiments. The expression of 67LR, a high-affinity LN-binding protein, was analysed by Northern blotting and FACS analysis. All human MM cell lines and the murine 5T2MM cells expressed 67LR. 67LR is expressed by several normal cell types, as well as cancer cells of different origin, including B cell lymphoma (Carbone et al, 1995), but its expression on MM cells has not been reported so far. In vitro migration experiments demonstrated that LN stimulated the chemotactic migration of HMCL and murine 5T2MM cells. Previous studies revealed that the action of LN appeared through binding to 67LR with a sequence of 9 amino acids Cys-Asp-Pro-Gly-Tyr-Ile-Gly-Ser-Arg (CDPGYIGSR) (Situ et al, 1984). A synthetic nonapeptide (peptide 11), derived from this aminosequence was found to be able to compete with LN for binding to 67LR (Graf et al, 1987b). In vivo, it reduces the formation of experimental lung and osteolytic metastasis (Iwamoto et al, 1987, 1996; Nakai et al, 1992) and inhibits human pre-B leukaemic cell growth and dissemination to organs in SCID (Yoshida et al, 1999). We evaluated the effect of this synthetic nonapeptide on MM cell migration and observed that peptide 11 reduced MM cell migration to LN. In addition, we could demonstrate that the migration of MM cells could be inhibited by a blocking polyclonal antiserum against 67LR (Clement et al, 1990). These data allow us to conclude that 67LR plays an important role in the motility-promoting activity of LN on MM cells. The polyclonal antiserum against 67LR could not induce a complete inhibition of MM cell migration to LN indicating that other receptors might influence this motility process as well. In a previous study, Shibayama et al (1995) demonstrated that the migration of 2 HMCL (FR4ds and OPM-1 ds) to LN could be partially inhibited by a blocking antibody against the common chain of β1 integrins. This may indicate that the overall migration of MM cells to LN might be mediated by multiple receptors. Freshly isolated MM plasma cells from patient BM samples were also found to express 67LR but at a much lower level. Interestingly, 67LR expression could be enhanced when primary MM cells were co-cultured with a human BM endothelial cell line, whereas 67LR expression remained unchanged after contact with BM stromal fibroblasts (data not shown). Moreover, in vitro migration experiments with primary MM cells revealed an enhanced migration response to laminin when Transwell[R] filters of the migration system were coated with endothelial cells. This could indicate that 67LR is temporally up-regulated at the moment that MM cells adhere to and migrate through the endothelium while receptor expression decreases when tumour cells reside as fully matured plasma cells in the BM compartment. Accordingly, HMCL might express more 67LR because they reflect a more immature tumour stage than fully matured plasma cells. It was demonstrated that 67LR was co-localised with alpha-actin and vinculin, 2 structural cell proteins (Massia et al, 1993) suggesting a direct role for 67LR in cell motility.

To determine at which level LN-67LR interactions also influence the in vivo homing of MM cells, we used the murine 5T2MM model. This model has been intensively characterised and was found to express very similar pathological and biological features as human MM (Vanderkerken et al, 1996, 1997). In a recent study, we could demonstrate that 5T2MM cells in vivo selectively home to BM and liver and not to other organs. Moreover, we found that 5T2MM cells adhere selectively to BM, spleen and liver and not to lung endothelial cells (Vanderkerken et al, 2000). In the present study, we demonstrated that 5T2MM cells express 67LR and migrate in vitro to LN and peptide 11. When radiolabelled 5T2MM cells were mixed with peptide 11 and co-injected into syngeneic mice, the proportion of tumour cells which accumulated in the BM after 18 hours was significantly decreased in peptide 11-treated animals. This observation indicates that interaction between 67LR and LN is at least partially involved in the in vivo migration of 5T2MM cells through BM endothelium. Because of

*© 2001 Cancer Research Campaign*

the broad expression of LN in extracellular matrices throughout the body, it is clear that this molecule itself can not be the only factor that determines the specificity of MM cell homing. It can be assumed that several adhesion mechanisms and chemotactic signals co-act to enhance the selectivity of tumour cell migration. Moreover, the restricted localisation of MM cells in the BM might also relate to the presence of a unique combination of growth and survival factors, which is not available at other tissue sites. In conclusion, we demonstrated that MM cells express the high-affinity 67 kD LN receptor and that this receptor is involved in LN-mediated cell migration. It is likely that 67LR is involved during the extravasation of MM cells to the bone marrow. The homing of myeloma cells to the bone marrow is believed to be a multistep process, involving the action of various adhesion molecules, chemotactic signals and metalloproteases. Interference with one of the key steps in this process (like migration to laminin) might be an important therapeutical tool to reduce tumour dissemination.

### ACKNOWLEDGEMENTS

The authors wish to thank Nicole Arras and Wim Renmans for excellent technical assistance, Dr V Castronovo (Laboratory of Metastasis Research, University of Liège, Belgium) for the generous gift of the monoclonal antibodies against 67LR and the cDNA probe recognising mRNA of the 37 kD precursor for 67LR, Dr H Kleinman (National Institute of Dental and Craniofacial Research, National Institute of Health, Bethesda, USA) for generously providing the blocking polyclonal antiserum against 67LR and Dr A Dräger (Vrije Universiteit Amsterdam, The Netherlands) for giving the 4LHBMEC cell line. This work was supported by grants of the Fund for Scientific Research, Flanders (FWO-Vlaanderen), Belgium and the International Myeloma Foundation (Brian Novis Research Grant 1999 and 2000 to Ivan Van Riet). Isabelle Vande Broek is a research assistant and Karin Vanderkerken is a post-doctoral fellow of the FWO-Vlaanderen.

### REFERENCES

Asosingh K, Radl J, Van Riet I, Van Camp B and Vanderkerken K (2000) The 5TMM series, a useful in vivo mouse model of human multiple myeloma. *Hematol Journal* **1**: 351–356

Bakkus M, Heirman C, Van Riet I, Van Camp B and Thielemans K (1992) Evidence that multiple myeloma Ig heavy chain VDJ genes contain somatic mutations but show no intraclonal variation. *Blood* **80**: 2326–2335

Butcher EC and Picker LJ (1996) Lymphocyte homing and homeostasis. *Science* **272**: 60–66

Carbone A, Gloghini A, Colombatti A, Castronovo V and Menard S (1995) Expression of the monomeric 67-kd laminin-binding protein in human lymphomas as defined by MLuC5 monoclonal antibody and paraffin section immunohistochemistry. *Hum Pathol* **26**: 541–546

Clement B, Segui-Regal B, Savagner P, Kleinman HK and Yamada Y (1990) Hepatocyte attachment to laminin is mediated through multiple receptors. *J Cell Biol* **110**: 185–192

D'Errico A, Liotta LA, Castronovo V, Stetler-Stevenson WG and Grigioni WF (1991) Augmentation of type IV collagenase, laminin receptor and Ki67 proliferation antigen associated with human colon, gastric and breast cancer progression. *Mod Pathol* **4**: 239–246

Durie BG and Salmon SE (1975) A clinical staging system for multiple myeloma. Correlation of measured myeloma cell mass with presenting clinical features, response to treatment, and survival. *Cancer* **36**: 842–854

Graf J, Iwamoto Y, Sasaki M, Martin GR, Kleinman HK, Robey FA and Yamada Y (1987a) Identification of an amino acid sequence in laminin mediating cell attachment, chemotaxis, and receptor binding. *Cell* **48**: 989–996

Graf J, Ogle RC, Robey FA, Sasaki M, Martin GR, Yamada Y and Kleinman HK (1987b) A pentapeptide from the laminin B1 chain mediates cell adhesion and binds the 67.000 laminin receptor. *Biochemistry* **26**: 6896–6900

Heino J (1996) Biology of tumor cell invasion: interplay of cell adhesion and matrix degradation *Int J Cancer* **65**: 717–722

Iwamoto Y, Robey FA, Graf L, Sasaki M, Kleinman H, Yamada H and Martin GR (1987) YIGSR, a synthetic laminin pentapeptide, inhibits experimental metastasis formation. *Science* **23**: 1132–1134

Iwamoto Y, Nomizu M, Yamada Y, Ito Y, Tanaka K and Sugioka Y (1996) Inhibition of angiogenesis, tumour growth and experimental metastasis of human fibrosarcoma cells HT1080 by a multimeric form of the laminin sequence Tyr-Ile-Gly-Ser-Arg (YIGSR). *Br J Cancer* **73**: 589–595

Kim WH, Lee BL, Jun SH, Song SY and Kleinman HK (1998) Expression of 32/67 kDa laminin receptor in laminin adhesion-selected colon cancer cell lines. *Br J Cancer* **77**: 15–20

Klominek J, Robert KH and Sundqvist KG (1993) Chemotaxis and haptotaxis of human malignant mesothelioma cells: effects of fibronectin, laminin, type IV collagen and an autocrine motility factor-like substance *Cancer Res* **53**: 4376–4382

Liotta LA (1986) Tumor invasion and metastasis – role of the extracellular matrix *Cancer Res* **46**: 1–7

Ludwig H, Meran J and Zojer N (1999) Multiple myeloma, an update on biology and treatment. *Ann Oncology* **10**: (suppl 6): 31–43

Malinda KM and Kleinman HK (1996) The laminins *Int J Biochem Cell Biology* **28**: 957–959

Malinoff HL and Wicha MS (1983) Isolation of a cell surface receptor protein for laminin from murine fibrosarcoma cells. *J Cell Biol* **96**: 1475–1479

Massia SP, Sekhar SR and Hubbel JA (1993) Covalently immobilized laminin peptide Tyr-Ile-Gly-Ser-Arg (YIGSR) supports cell spreading and co-localization of the 67-kilodalton laminin receptor with alfa-actinin and vinculin. *J Biol Chem* **268**: 8053–8059

Nakai M, Mundy GR, Williams PJ, Boyce B and Yoneda T (1992) A synthetic antagonist to laminin inhibits the formation of osteolytic metastasis by human melanoma cells in nude mice. *Cancer Res* **52**: 5395–5399

Nilson K (1971) Characteristics of established myeloma and lymphoblastoid cell lines derived from an E myeloma patient. *Int J Cancer* **7**: 380–396

Okuno Y, Takahaschi T, Suzuki A, Ichiba S, Nakamura K and Imura H (1991) Establishment and characterization of four myeloma cell lines which are responsive to interleukin-6 for their growth. *Leukemia* **5**: 585–591

Radl J, De Glopper E, Schuit HRF and Zucher C (1979) Idiopathic paraproteinemia II. Transplantation of the paraprotein-producing clone from old to young C57BL/KaLwRij mice. *J Immunol* **122**: 609

Ramos DM, Cheng YF and Kramer RH (1991) Role of laminin-binding integrin in the invasion of basement membrane matrices by fibrosarcoma cells. *Invasion Metastasis* **11**: 125–138

Roehm NW, Rodgers GH, Hatfield SM and Glasebrook AL (1991) An improved colorimetric assay for cell proliferation and viability utilizing the tetrazolium salt XTT. *J Immunol Methods* **142**: 257–265

Satoh K, Narumi K, Abe T, Sakai T, Kikuchi T, Tanaka M, Shimo-Oka T, Uchida M, Tezuka F, Isemura M and Nukiwa (1999) Diminution of 37-kDa laminin binding protein expression reduces tumour formation of murine lung cancer cells. *Br J Cancer* **80**: 1115–1122

Shibayama H, Tagawa S, Hattori H, Inoue R, Katagiri S and Kitani T (1995) Laminin and fibronectin promote the chemotaxis of human malignant plasma cell lines. *Blood* **86**: 719–725

Situ R, Lee EC, McCoy JP and Varani J (1984) Stimulation of tumour cell motility by laminin. *J Cell Sci* **70**: 167–176

Spessotto P, Yin Z, Magro G, Deutzmann R, Chiu A, Colombatti A and Perris R (2001) Laminin isoforms 8 and 10 are primary components of the subendothelial basement membrane promoting interaction with neoplastic lymphocytes *Cancer Res* **61**: 339–347

Van Riet I, Heirman C, Lacor P, De Waele M, Thielemans K and Van Camp B (1989) Detection of monoclonal B lymphocytes in bone marrow and peripheral blood of multiple myeloma patients by immunoglobulin gene rearrangement studies. *Br J of Haematol* **73**: 289–295

Van Riet I, De Greef C, Aharchi F, Woischwill C, De Waele M, Lacor P, Schots R and Van Camp B (1997) Establishment and characterisation of a human stroma-dependent multiple myeloma cell line (MM5.1) and its stroma-independent variant (MM5.2). *Leukemia* **11**: 284–293

Van Riet I, Vanderkerken K, De Greef C and Van Camp B (1998) Homing behaviour of the malignant clone in multiple myeloma. *Med Oncol* **15**: 154–164

Vanderkerken K, Goes E, De Raeve H, Radl J and Van Camp B (1996) Follow-up of bone osteolytic lesions in an experimental multiple myeloma mouse model: description of an in vivo technique using radiography dedicated for mammography. *Br J Cancer* **73**: 1463–1465

 *© 2001 Cancer Research Campaign*

Vanderkerken K, De Raeve H, Goes E, Van Meirvenne S, Radl J, Van Riet I, Thielemans K and Van Camp B (1997) Organ involvement and phenotypic adhesion profile of 5T2 and 5T33 myeloma cells in the C57/KaLwRij mice. *Br J Cancer* **76**: 451–460

Vanderkerken K, De Greef C, Asosingh K, Arteta B, De Veerman M, Vande Broek I, Van Riet I, Kobayashi M, Smedsrod B and Van Camp B (2000) Selective in vivo homing pattern of 5T2 multiple myeloma cells in the C57BL/KalwRij mouse. *Br J Cancer* **82**: 953–959

Vracko R (1974) Basal lamina scaffold-anatomy and significance for maintenance of orderly tissue structure. *Am J Pathol* **77**: 314–346

Wewer U, Taraboletti G, Sobel ME, Albrechtsen R and Liotta A (1987) Role of laminin receptor in tumor cell migration. *Cancer Res* **47**: 5691–5698

Yoshida N, Ishii E, Nomizu M, Yamada Y, Mohri S, Kinukawa N, Matsuzaki A, Oshima K, Hara T and Miyazaki S (1999) The laminin-derived peptide YIGSR (Tyr-Ile-Gly-Ser-Arg) inhibits human pre-B leukaemic cell growth and dissemination to organs in SCID mice. *Br J Cancer* **80**: 1898–1904

Zigmond SH and Hirsch JG (1973) Leukocyte locomotion and chemotaxis. *J Exp Med* **137**: 387–410

© 2001 Cancer Research Campaign
